# Mediation Effects of Biobehavioral Factors in a Trial of Pharmacotherapy and Intensive Cessation Counseling for People with HIV Who Smoke Cigarettes in Nairobi, Kenya

**DOI:** 10.1007/s10461-025-04968-5

**Published:** 2025-12-10

**Authors:** Angela A. Omanya, Jonathan Shuter, Emily Koech, Sylvia Ojoo, Wendy Potts, Lan Li, Christopher W. Kahler, Seth S. Himelhoch

**Affiliations:** 1 Center for International Health, Education and Biosecurity-Kenya (CIHEB-Kenya), Nairobi, Kenya; 2 Albert Einstein College of Medicine, 1300 Morris Park Avenue, Bronx, NY 10461 USA; 3 University of Maryland School of Medicine, Baltimore, USA; 4 Georgetown School of Medicine, Washington, USA; 5 Center for Alcohol and Addiction Studies, Brown University School of Public Health, Providence, USA; 6 University of Chicago Pritzker School of Medicine, Chicago, USA

**Keywords:** HIV, Tobacco, Smoking, Mediation, Self-efficacy

## Abstract

There is growing recognition of the important health risks of tobacco use in people with HIV (PWH). Multiple randomized controlled trials have tested cessation treatments in this population, but little is known about factors that mediate successful quitting. We conducted a randomized, placebo-controlled 2 × 2 factorial design trial of a behavioral intervention (Positively Smoke Free [PSF] one-on-one counseling) vs. brief advice to quit ± bupropion vs. placebo in PWH who smoked cigarettes in Nairobi, Kenya. Abstinence from cigarettes was assessed by self-report and exhaled carbon monoxide (ECO). We conducted pre-planned analyses of putative mediators of the effects of bupropion (i.e. craving, withdrawal, negative affect) and of PSF counseling (i.e. abstinence self-efficacy, decisional balance, and loneliness) at 12-weeks on biochemically-confirmed abstinence at 36-weeks. 269 participants were included in the final analytic cohort (mean age = 42.7 years, 70.3% male, smoking a mean of 10.6 cigarettes per day). The biochemically verified abstinence rate at 36-weeks was 24.2%. PSF counseling increased abstinence self-efficacy and reduced loneliness significantly more than brief advice to quit at 12-weeks. Mediation analyses suggested a mediating effect of change in self-efficacy at 12-weeks in the relationship of PSF to abstinence at 36-weeks. None of the putative mediators demonstrated a significant mediation effect of bupropion on quitting. These results indicate that self-efficacy was one mechanism through which PSF counseling, but not bupropion, increased smoking abstinence among PWH who smoked cigarettes in Nairobi, Kenya.

Trial Registration: NCT02460900

**Supplementary Information:**

The online version contains supplementary material available at 10.1007/s10461-025-04968-5.

## Introduction

People with HIV (PWH) smoke cigarettes at significantly higher rates than the general population, and they are less likely to quit [1, 2]. Although much of the research on tobacco use among PWH has been performed in high-income countries (HIC), these disparities appear to hold true in low- and middle-income countries (LMIC), where most of the world’s PWH reside [3]. 

With the widening access to antiretroviral treatment, PWH are now losing more life years to smoking than to viral infection [4, 5]. This reality has prompted significant interest in the optimization of smoking cessation efforts for PWH. Since quit rates among PWH who smoke have been, overall, disappointing in trials of various behavioral and pharmacologic cessation therapies, some recent research has focused on identifying the mechanisms of action of interventions that have succeeded in promoting cessation in this highly vulnerable group [6–8]. In a recently published trial [9], we aimed to evaluate the effects of smoking cessation interventions for PWH living in Nairobi by using a two-by-two factorial design with behavioral and medication treatments each with a control/placebo arm. In this trial, both Positively Smoke Free (PSF) individual cessation counseling and bupropion, separately and in combination, were shown to be more effective than brief counseling or placebo. In the current study we report the results of pre-planned analyses assessing the mediation effects, or lack thereof, of several putative mediators of both PSF and bupropion on tobacco use outcomes.

The study protocol specified pre-planned mediation analyses exploring the presence or absence of mediating effects of a number of a priori selected variables that were hypothesized to function as mediators of the two interventions’ (bupropion and PSF) effects on the smoking cessation outcome. Earlier research has focused on negative affect and craving and withdrawal symptoms as possible mediators of bupropion’s effects on smoking behaviors in other populations [10, 11], and on abstinence self-efficacy as a mediator of counseling’s effects on smoking behaviors in PWH [6–8]. In the current study, for bupropion, mediators focused on craving, withdrawal, and depression, which are potential physiologic targets of bupropion’s action [10, 12], and mediators for PSF focused on the behavioral targets of PSF counseling, including abstinence self-efficacy, smoking decisional balance, and loneliness.

## Methods

We conducted a randomized, placebo-controlled trial of a pharmacologic (bupropion vs. visually identical placebo) and a behavioral intervention (Positively Smoke Free one-on-one counseling vs. brief advice to quit one-on-one counseling, referred to in the trial as “standard of care” or SOC) in a cohort of people with HIV who smoked cigarettes in Nairobi, Kenya between June 2020 and July 2023.

Full details of the study methodology and primary outcomes have been published in a prior paper [9]. In brief, we enrolled PWH who smoked cigarettes into a two-by-two factorial design trial that randomized participants into the following four conditions: (1) bupropion (12-weeks) + PSF (8 weeks); (2) bupropion (12-weeks) + Standard of Care (SOC), i.e., brief advice to quit; (3) placebo (12-weeks) + PSF (8 weeks); and (4) placebo (12-weeks) + SOC. The primary outcome was the exhaled carbon monoxide (ECO)-confirmed, 7-day point prevalence smoking abstinence (PPA) at 36-weeks. The trial was IRB approved and registered with ClinicalTrials.gov (NCT02460900).

At enrollment (0-weeks), 12-weeks, and 36-weeks, participants completed pencil and paper study interviews administered by study staff that queried a range of socio-behavioral domains, especially those relating to HIV infection and tobacco use.

The scales employed for putative bupropion effect mediators were the Tobacco Craving Questionnaire – Short Form (TCQ-SF) [13], the Minnesota Nicotine Withdrawal Scale – Short Form (MNWS-SF) [14], and the Center of Epidemiologic Studies Depression Scale – 10 item (CES-D-10) [15]. The scales employed for putative PSF effect mediators were the Smoking: Self-efficacy/Temptation – Short Form [16], the Smoking: Decisional Balance – Short Form [17], and the University of California Los Angeles Loneliness Scale – Short Form [18]. The details pertaining to these various scales may be found in Appendix 1. All were assessed at both 12 and 36-weeks.

For univariate analyses of change in continuous variables compared over two timepoints we used paired Student’s t-tests within each intervention condition. Analyses were also repeated using repeated measure ANOVA so that the time*bupropion*PSF interaction could be examined along with the bupropion*time and PSF*time effects; those results were consistent with the pre-registered *t*-test analyses and single mediation analyses reported below and are available in Supplemental Table 1.

We employed the methodology of MacKinnon et al. to conduct single mediation analyses [19]. The predictor variable was the intervention allocation, either bupropion or PSF, and the outcome variable was biochemically verified seven-day point prevalence abstinence at the 36-week timepoint. These analyses used an intention-to-treat approach and were indifferent to participants’ actual receipt of the assigned intervention. As noted in our prior paper, adherence to assigned interventions exceeded 95% in all groups [9]. Mediation analyses were only completed for putative mediators that demonstrated a significant association in the c-path of MacKinnon’s model [19]. For the prespecified putative mediator variables, a change score was calculated for each of them at the 12-week timepoint by subtracting the baseline value from the 12-week value. These mediator variables were pre-registered at the time of protocol entry on clinicaltrials.gov and may be accessed at https://cdn.clinicaltrials.gov/large-docs/27/NCT03342027/SAP_001.pdf. A series of mediation models were developed to examine the indirect effects of putative mediators on smoking abstinence. First, the total effect of the intervention on the cessation outcome (biochemically-confirmed abstinence at 36-weeks) controlling for the putative mediator’s value at baseline was assessed with logistic regression. Next, the a-path from the intervention to the putative mediator was assessed with linear regression, and the b-path from the putative mediator at 12-weeks to the outcome variable of abstinence at 36-weeks was assessed with logistic regression. The indirect effect of the intervention on smoking abstinence on the mediator was calculated by multiplying the a-path by the b-path. Confidence intervals for estimates were generated using 5,000 bootstrap replicates. The direct effect was the c’-path regressed on the predictor with the putative mediator variable.

From our prior paper [9], sex was identified as a potential confounder in the analyses on the smoking abstinence outcome. Therefore, we adjusted all mediation models by sex to evaluate if there were any substantial changes in the models. Mediation analyses were conducted using SAS Process Macro Version 4.3.

## Results

The analytic sample in the parent trial was comprised of 294 participants, and a summary of their clinical and sociodemographic profiles has been published elsewhere [9]. In the current study, 269 participants (91.5% of the parent trial cohort) contributed complete data at the 12-week timepoint and were thus included. Table 1 summarizes the sociodemographic characteristics of these 269 participants. Sixty-five participants (24.2%) had biochemically-verified abstinence at 36-weeks. The abstinence rates in each of the different study conditions are detailed in Table 1. Participants who were randomized to receive bupropion were more likely to quit than those randomized to receive placebo, OR = 3.02 (95% CI: 1.67–5.49, *P* = 0.0002), and participants who were randomized to receive PSF counseling were more likely to quit than those randomized to standard of care, OR = 2.43 (95% CI: 1.35–4.35, *P* = 0.002).

**Table 1:** Baseline demographics of the study sample stratified by study condition

	Total	PSF + Bupropion	PSF + Placebo	SOC + Bupropion	SOC + Placebo	
	(*N* = 269)	(*N* = 67)	(*N* = 67)	(*N* = 65)	(*N* = 70)	
Age in years, mean (SD)	42.7(10.4)	41.6(10.4)	40.7(10.2)	44.4(10)	44.3(10.4)	
Sex						
Male	189(70.3%)	48(71.6%)	47(70.1%)	43(66.2%)	51(72.9%)	
Female	80(29.7%)	19(28.4%)	20(29.9%)	22(33.8%)	19(27.1%)	
Race						
Asian	1(0.4%)	0(0.0%)	1(1.5%)	0(0.0%)	0(0.0%)	
African	268(99.6%)	67(100.0%)	66(98.5%)	65(100.0%)	70(100.0%)	
Education						
<12 years	234(87.6%)	57(86.4%)	57(85.1%)	58(90.6%)	62(88.6%)	
12 + years	33(12.4%)	9(13.6%)	10(14.9%)	6(9.4%)	8(11.4%)	
Employed						
Yes	239(89.5%)	58(86.6%)	62(93.9%)	57(89.1%)	62(88.6%)	
No	28(10.5%)	9(13.4%)	4(6.1%)	7(10.9%)	8(11.4%)	
Marital Status						
Married	136(50.7%)	29(43.3%)	33(49.3%)	39(60.9%)	35(50.0%)	
Single/Divorced/Widow/Separated	132(49.3%)	38(56.7%)	34(50.7%)	25(39.1%)	35(50.0%)	
Current housing status						
Stable	257(95.9%)	64(95.5%)	62(92.5%)	63(98.4%)	68(97.1%)	
Transitional/Homeless	11(4.1%)	3(4.5%)	5(7.5%)	1(1.6%)	2(2.9%)	
Years since HIV diagnosis, mean (SD)	8.5(6.1)	7.5(5.8)	9.4(6.3)	8.8(6.7)	8.5(5.7)	
Receiving antiretroviral therapy	268(100.0%)	67(100.0%)	67(100.0%)	64(100.0%)	70(100.0%)	
Age in years at first cigarette mean (SD)	20.9(7.5)	21(7.5)	20.4(6.0)	20.3(8.0)	21.9(8.4)	
Cumulative years of cigarette smoking, mean (SD)	21.8(11.4)	20.6(10.6)	20.3(10.6)	24.1(12.1)	22.3(12.0)	
Average cigarettes per day, mean (SD)	10.6(7.2)	10.3(6.7)	10.0(7.7)	10.8(7.7)	11.2(6.7)	
Fagerström Test of Cigarette Dependence (0–10), mean (SD)	4.5(2.3)	4.6(2.2)	4.5(2.2)	4.4(2.5)	4.5(2.2)	
Smoking abstinence at week 12	81(30.1%)	33(49.3%)	21(31.3%)	16(24.6%)	11(15.5%)	
Smoking abstinence at week 36	65(24.2%)	28(41.8%)	15(22.4%)	17(26.2%)	5(7.0%)	

*PSF* Positively Smoke Free, *SOC *Standard of care, *SD *standard deviation

Tables 2 and 3 present univariate analyses of changes in the putative mediator variable scores between baseline and 12-weeks for bupropion vs. placebo and for PSF vs. SOC. There were statistically significant reductions in tobacco craving (df = 127, t = 11.79, *P* < 0.0001 for bupropion; df = 130, t = 9.99, *P* < 0.0001 for placebo), withdrawal symptoms (df = 127, t = 6.71, *P* < 0.0001 for bupropion; df = 130, t = 6.24, *P* < 0.0001 for placebo), and depression score (df = 127, t = 3.55, *P* = 0.0005 for bupropion; df = 130, t = 3.79, *P* = 0.0002 for placebo) in both the bupropion and placebo arms. There were no significant differences in the change of any of these three variables from baseline to 12-weeks in the bupropion arm as compared to placebo arm. There were statistically significant increases in self-efficacy (df = 128, t = 18.06, *P* < 0.0001 for PSF; df = 129, t = 13.82, *P* < 0.0001 for SOC) and decisional balance–pro scores (df = 128, t = 8.36, *P* < 0.0001 for PSF; df = 129, t = 6.08, *P* < 0.0001 for SOC) and statistically significant reductions in loneliness (df = 128, t = 5.88, *P* < 0.0001 for PSF; df = 129, t = 3.15, *P* = 0.002 for SOC) and decisional balance–con (df = 128, t=−3.31, *P* = 0.0012 for PSF; df = 129, t=−3.32, *P* = 0.0012 for SOC) scores in both the PSF and SOC arms. Those participants who received PSF as compared to SOC had a significantly greater increase in self-efficacy (df = 257, t = 3.28, *P* = 0.001) and decrease in loneliness scores (df = 257, t = 1.99, *P* = 0.048).

**Table 2:** Means and standard deviations (SD) of hypothesized mediators of bupropion effect at baseline and 12-weeks (*N* = 269)

Mediator	Baseline		12-weeks	
	Placebo	Bupropion	Placebo	Bupropion
	Mean (SD)	Mean (SD)	Mean (SD)	Mean (SD)
Tobacco Craving Total	3.1 (1.8)	3.4 (1.9)	1.6 (0.9)***	1.4 (1.1)***
Minnesota Withdrawal Symptoms Total	0.6 (0.6)	0.6 (0.5)	0.3 (0.5)***	0.2 (0.5)***
CES-D 10	8.4 (3.2)	8.2 (3.2)	7.2 (2.5)***	7.1 (2.5)***

Outcome			Placebo	Bupropion
			N (%)	N (%)
Biochemically-verified abstinence at 36-weeks				
Yes			20(14.6%)	45(34.1%)
No			117(83.7%)	87(65.9%)

The changes that are statistically significant: *P < 0.05, **P < 0.01, ***P < 0.001. Bold font represents paired sample comparisons of 12-week vs. baseline data for the placebo and bupropion arms, and P-values are calculated for the within-group comparisons at baseline and 12-weeks of means for placebo and bupropion

CES-D-10 = Center of Epidemiologic Studies Depression Scale 10-item

**Table 3:** Means and standard deviations (SD) of hypothesized mediators of PSF effects at baseline and 12-weeks (*N* = 269)

Mediator	Baseline		12-weeks	
	SOC	PSF	SOC	PSF
	Mean (SD)	Mean (SD)	Mean (SD)	Mean (SD)
Self-efficacy score	3.7 (1.0)	3.9 (0.9)	2.2 (1.1)***	1.8 (1.1)***
Loneliness score	14.2 (6.4)	15.4 (7.0)	12.5 (5.1)**	12.0 (3.8)***
Decisional Balance Pro	5.8 (3.5)	6.0 (3.5)	3.8 (1.9)***	3.3 (1.3)***
Decisional Balance Con	11.7 (3.2)	11.3 (3.2)	13.0 (3.0)**	12.6 (3.5)**

Outcome			SOC	PSF
			N (%)	N (%)
Biochemically-verified abstinence at 36-weeks				
Yes			22 (16.3%)	43 (32.1%)
No			113 (83.7%)	91 (67.9%)

The changes that are statistically significant: *P < 0.05, **P < 0.01, ***P < 0.001. Bold font represents paired sample comparisons of 12-week vs. baseline data for the PSF and SOC arms, and P-values are calculated for the within-group comparisons of means at baseline and 12-weeks for PSF and SOC

PSF = Positively Smoke Free, SOC = Standard of care, SD = standard deviation

**Fig. 1:**
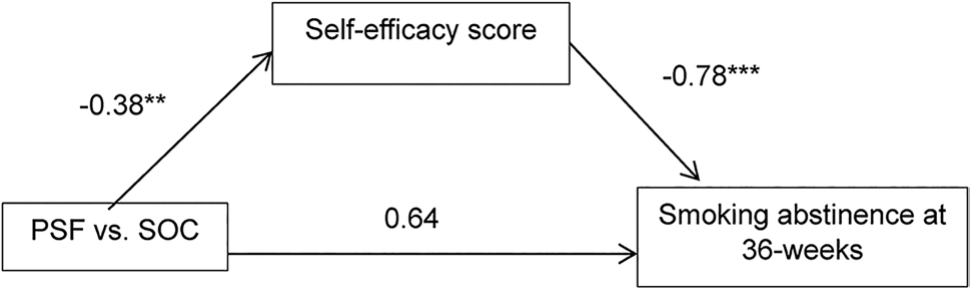
Mediation diagram depicting the mediation effect of self-efficacy score on the relationship between assignment to the PSF condition and abstinence at 36-weeks. Mediation results of PSF vs SOC on smoking abstinence at 36-weeks with change scores of self-efficacy score from baseline to 12-weeks. lower self-efficacy score connotes higher self-efficacy. *PSF* Positively Smoke Free, *SOC *standard of care. **p < 0.01, ***p < 0.001

The results of the mediation analyses assessing the effects of changes in the tobacco craving, withdrawal, and depression scores on the overall effect of bupropion on 36-week abstinence, and assessing the effects of changes in self-efficacy, decisional balance, and loneliness scores on the overall effect of PSF on 36-week abstinence are listed in Table 4. In summary, the only putative mediator found to exert a mediation effect of either of the cessation interventions on 36-week abstinence was self-efficacy, with weakening of the direct association of PSF with the abstinence outcome (*P* = 0.002) when change in self-efficacy at 12-weeks was included in the model (*P* = 0.0439). When we incorporated sex into the mediation model, it did not result in any significant changes in the analytic outcomes. Figure 1 is the mediation analysis diagram depicting the mediating relationship between PSF, self-efficacy, and 36-week abstinence.

**Table 4:** Putative mediator variables on the relationship between intervention groups at 12-weeks and quit rate (*n* = 269) at 36-weeks

Mediators (Change score)	Indirect effect^a^ (ab path)				Effect of intervention on mediator (a path)				Effect of mediator on outcome (b path)				Adjusted Direct effect (c path)			
	Value	p	LCL	UCL	Value	p	LCL	UCL	Value	p	LCL	UCL	Value	p	LCL	UCL
**Bupropion**																
Tobacco Craving Total	0.13	0.2346	−0.05	0.44	−0.17	0.1551	−0.41	0.07	−0.73	0.0086	−1.27	−0.19	0.98	0.0020	0.36	1.61
Minnesota Withdrawal Symptoms Total	0.005	0.9454	−0.22	0.23	−0.004	0.9399	−0.12	0.11	−1.21	0.0310	−2.32	−0.11	1.12	0.0004	0.50	1.74
CES-D 10 total	−0.00001	0.9999	−0.08	0.12	0.0001	0.9998	−0.59	0.59	−0.10	0.1940	−0.25	0.05	1.10	0.0004	0.49	1.71
**PSF**																
Self-efficacy: Total	0.30	0.0207	0.08	0.63	−0.38	0.0041	−0.64	−0.12	−0.78	0.0000	−1.16	−0.41	0.64	0.0439	0.02	1.27
Loneliness score	0.03	0.5016	−0.02	0.20	−0.75	0.1570	−1.79	0.29	−0.04	0.3507	−0.12	0.04	0.95	0.0056	0.25	1.45
Decisional Balance pro	0.08	0.2998	−0.03	0.61	−0.51	0.0135	−0.91	−0.11	−0.16	0.2189	−0.41	0.09	0.81	0.0090	1.20	1.42
Decisional Balance con	0.01	0.6172	−0.03	0.09	−0.33	0.4186	−1.12	0.47	−0.04	0.3130	−0.13	0.04	0.89	0.0036	0.29	1.49

Results are expressed in log-odds values. PSF = Positively Smoke Free. CES-D 10 = Center of Epidemiologic Studies Depression Scale 10-item. LCL = lower 95% confidence interval. UCL = upper 95% confidence interval

^a^Confidence intervals (CIs) resulted from 5,000 bootstrap replicates

## Discussion

In this paper we evaluated a group of variables that we hypothesized to serve as potential mediators of the effects of bupropion and Positively Smoke Free counseling on smoking cessation in a cohort of PWH in Nairobi, Kenya who were actively smoking cigarettes at the time of study enrollment.

The univariate analyses presented in Tables 2 and 3 demonstrated significant changes in all putative mediators between baseline and the 12-week timepoint, both in the intervention and control arms. It is not unusual to observe salutary health effects in clinical trials related to trial participation regardless of treatment allocation [20]; this, in fact, is a main justification for the inclusion of control arms in clinical research. In our prior work, we have shown that clinical trial participants reduced their average daily cigarette consumption significantly in both intervention and control arms [21]. Such non-specific effects may have been accentuated by social desirability bias since the questionnaires were directly administered to the participants by the study staff. Consistent with the study hypotheses, changes in self-efficacy and loneliness were greater in the PSF arm than in the SOC arm.

Prior research has evaluated tobacco craving, withdrawal symptoms, and negative affect as potential mediators of bupropion’s effect on smoking cessation [10, 12]. Earlier studies have suggested a mediating effect of self-efficacy and decisional balance in the promotion of cessation in PWH who smoke cigarettes [6–8]. Since we have previously shown the high prevalence of loneliness among PWH [22], and others have implicated loneliness as a mediator of the effects of social support on health behaviors [23], including smoking, we opted to include loneliness in our list of putative mediators.

Bupropion is an antidepressant agent that acts as an inhibitor of both dopamine and norepinephrine uptake. Its chemical activity in the synaptic cleft is multifold and complex, and its activity in promoting smoking cessation has been attributed to its effects on dopamine and norepinephrine uptake as well as an antagonistic effect on the interaction of nicotine with the post-synaptic acetylcholine nicotinic receptor [24]. Meta-analytic data that include 71 randomized controlled trials and more than 14,000 participants have established bupropion as a drug that is effective in promoting smoking cessation [25]. Our research adds to this evidence base, and we are not aware of any prior randomized controlled trial to demonstrate the efficacy of this agent for PWH who smoke cigarettes. Bupropion is purported to exert its effect by reducing withdrawal symptoms during cessation attempts [26], and some studies have also suggested that it supports cessation by reducing negative affect [10]. 

Allocation to the bupropion versus the placebo condition was associated with 3.02 times the odds of quitting at 36-weeks (95% CI: 1.67–5.49, *P* = 0.0002). However, mediation analyses did not support the role of changes in tobacco withdrawal, tobacco craving, or depression score as mediators of this effect. This is similar to findings reported by other investigators [12]. 

Positively Smoke Free (PSF) is an intensive multisession behavioral intervention targeting PWH who smoke cigarettes that has been delivered in clinical trials to a range of populations utilizing various formats including in-person group therapy, in-person individual therapy, online therapy with an online support community, and via a mobile app [8, 27–30]. Most of these trials have demonstrated higher quit rates in the intervention than in the control arm, and three of them, including the current study, have demonstrated higher quit rates at or beyond the critical six-month timepoint [31, 32]. PSF content is tailored to address concerns that are common among PWH who smoke, and it attempts to bolster abstinence self-efficacy by anticipating high-temptation situations and planning strategies to either avoid them or to resist the urge to smoke when such situations arise. It also emphasizes the role of social support in successful quitting and the role of decisional balance, i.e. weighing the costs and benefits of each decision to light up a cigarette.

Allocation to the PSF condition versus a single session of brief advice to quit was associated with 2.43 times the odds of quitting at 36-weeks (95% CI: 1.35–4.35, *P*= 0.002). Similar to our prior work in a US population, and the results reported by other investigators as well [6–8], we found that those allocated to PSF counseling had significantly greater increases in self-efficacy at 12-weeks and that the changes in self-efficacy mediated the effect of PSF on biochemically- verified abstinence at the 36-week timepoint. We did not find that sex affected the mediation relationship in a substantive way. Neither changes in loneliness score nor changes in decisional balance met criteria for the mediation effect.

The observation that none of the variables met criteria for full mediation effects on cessation at the 36-week timepoint suggests that changes in the variables in the early phase of quit attempts may be critical to affecting the initial quit, and that other factors may be at play in sustaining abstinence over longer time periods. However, it is notable that the effect of allocation to the PSF condition on change in self-efficacy at 12-weeks was still significantly associated with abstinence at 36-weeks, albeit in the absence of full mediation in the formal mediation analysis. This does suggest that the behavioral effect of PSF counseling may help to sustain the quit through the critical 24-week timepoint.

Our study has several limitations that are worthy of mention. The trial was conducted in Nairobi, Kenya, and although similar mediation patterns have been shown in other regions [6–8], our findings are not necessarily generalizable to other geographic locations or communities. Although this was one of the larger studies of tobacco cessation strategies conducted in PWH, we may not have been adequately powered to detect small mediation effects. The restriction of data collection to three timepoints over 36 weeks did not permit the temporal granularity and precision that other techniques such as ecological momentary assessment can provide [12]. Notwithstanding these limitations, we believe that our findings are persuasive, they are aligned with the findings of earlier trials conducted in other areas of the world, and they advance the science of tobacco control in PWH.

Multiple studies have now shown that self-efficacy serves a mediating role in the effect of behavioral therapy on successful smoking cessation in PWH who smoke cigarettes [6–8]. These data are derived from samples in different geographic regions throughout the US, and now we report similar findings in a cohort of PWH who smoke cigarettes in Nairobi. Fortifying and bolstering participants’ self-efficacy to resist the temptation to smoke in various situations should be a central focus of all behavioral cessation interventions that target this vulnerable population.

## Supplementary Information

Below is the link to the electronic supplementary material.

Supplementary material 1 (DOCX 26.2 kb)

## Data Availability

The dataset utilized for this research will be shared on reasonable request to the authors.

## Appendix

### Additional Information on Measures

*Fagerström Test of Cigarette Dependence*: A 6-item measure evaluating tobacco dependence. A total score ranges from 0 to 10 with a higher score representing more physical dependence on nicotine.

*Tobacco Craving Questionnaire (TCQ-SF)*: A 12-item, multidimensional measure that assesses craving. The four dimensions include emotionality, expectancy, compulsivity and purposefulness. The total mean scores can each range from 1 to 7, with a higher mean score representing more craving. Each dimension can be scored with a mean score range from 1 to 7. A higher score indicates higher domain-based craving symptoms.

*Minnesota Nicotine Withdrawal Scale – Short Form (MNWS-SF)*: An 8-item measure that assesses tobacco withdrawal. Scores for each question can range from 0 to 4, and the total mean score can range from 0 to 4, with a higher score representing more severe withdrawal symptoms.

*Smoking Self-efficacy/Temptation Short Form*: A 9-item, multidimensional scale assessing temptation to smoke in various situations. The three dimensions include negative affect, social/positive, and habitual/craving. Total mean scores for smoking temptation can range from 1 to 5, with higher scores representing greater temptation to smoke and *lower abstinence self-efficacy*. Each dimension can be scored with a separate mean score ranging from 1 to 5. A higher score indicates higher dimensional based temptation and *lower abstinence self-efficacy*.

*Smoking Decisional Balance Questionnaire Short Form*: A 6-item scale measuring the participants’ opinion regarding the “pros” & “cons” of smoking. The measure reports the “pros” and “cons” of continuing smoking separately. Scoring for the “pros” of continuing smoking can range from a mean score of 1–5, with a higher score representing greater importance of the “pros of continuing smoking.” Scoring for the “cons” of continuing smoking can range from a mean score of 1–5, with a higher mean score representing greater importance of the “cons of continuing smoking.”

*UCLA Loneliness Scale Short Form*: A 10-item measure of loneliness. Individual items are scored from 1 to 4 with higher scores indicating more frequent feelings of loneliness. Total score can range from 10 to 40. The scores can be interpreted as follows: 10–17-low degree of loneliness; 18–24-moderate degree of loneliness; 25–32-moderately high degree of loneliness; and 33–40-high degree of loneliness.

*Center for Epidemiologic Studies Depression Scale – 10-Item*: A 10-item measure that evaluates symptoms of depression over the past week. Scores range from 0 to 30, with higher scores indicating greater depressive symptoms.
