# Supplementary material for: Mediation Effects of Biobehavioral Factors in a Trial of Pharmacotherapy and Intensive Cessation Counseling for People with HIV Who Smoke Cigarettes in Nairobi, Kenya
Source: AIDS Behav. 2025 Dec 10;30(5):1498–505. doi: 10.1007/s10461-025-04968-5 (PMC13014530; doi:10.1007/s10461-025-04968-5)
Supplement: Supplementary file 1 — Supplementary material 1 (DOCX 26.2 kb) [file 10461_2025_4968_MOESM1_ESM.docx]

**Supplemental Table 1**. Mixed-effects ANOVA analyses.
